# Effects of mind-body exercise on balance function in pre-frail and frail older adults: a systematic review and meta-analysis

**DOI:** 10.3389/fragi.2026.1915136

**Published:** 2026-09-08

**Authors:** Wangyouyi Chen, Yiwen Bai, Liuling Song

**Affiliations:** 1 School of Rehabilitation Medicine, Shanghai University of Traditional Chinese Medicine, Shanghai, China; 2 Department of Physical Education, Sanda University, Shanghai, China

**Keywords:** balance function, frailty, meta-analysis, mind-body exercise, older adults, pre-frailty

## Abstract

**Background:**

Frailty and pre-frailty are common geriatric conditions associated with reduced physiological reserve and a higher risk of falls. Mind–body exercises such as Tai Chi, Baduanjin, and Yoga are widely recommended as low-intensity interventions, while their specific effects on different aspects of balance remain unclear.

**Objective:**

This systematic review and meta-analysis aimed to evaluate the effects of mind–body exercise on overall, dynamic, and static balance in pre-frail and frail older adults and to examine whether frailty stage and exercise characteristics influence outcomes.

**Methods:**

Our literature search was conducted in PubMed, Web of Science, Cochrane Library, Embase, CNKI, Wanfang Data, and VIP Databases up to March 2026. Randomized controlled trials comparing mind–body exercise with usual care, health education, or no intervention in pre-frail or frail older adults were included. Pooled effect sizes were calculated as standardized mean differences (SMDs) with 95% confidence intervals (CIs) using random-effects models. Prespecified subgroup analyses were performed according to frailty stage, exercise frequency, session duration, and intervention length.

**Results:**

Ten RCTs with 12 comparisons were included. Mind–body exercise showed significant improvements in overall balance (SMD = 0.54, 95% CI 0.17–0.91), dynamic balance (SMD = −1.08, 95% CI −1.41 to −0.76), and static balance (SMD = 1.36, 95% CI 0.43–2.30). Exploratory subgroup analyses suggested that interventions delivered 2–4 times weekly, lasting 31–60 min per session, and continuing for at least 13 weeks were associated with relatively more consistent effects; however, these findings should be interpreted cautiously because they were derived from indirect subgroup comparisons rather than formal dose–response analyses.

**Conclusion:**

Mind–body exercise appears to be an effective and practical strategy for improving balance in pre-frail and frail older adults, particularly when implemented early in the frailty trajectory.

## 1 Introduction

Frailty has been increasingly conceptualized as a multidimensional clinical condition characterized by reduced physiological reserve and impaired ability to maintain homeostasis in response to stressors. Rather than representing an inevitable consequence of aging, it is now understood as a dynamic state influenced by lifestyle, disease burden, and environmental exposures. Large epidemiological studies have consistently demonstrated that frailty is strongly associated with adverse outcomes, including falls, disability, hospitalization, and mortality, highlighting its relevance in both clinical practice and public health planning (Rockwood and Mitnitski, 2007; Morley et al., 2013; Walston et al., 2006).

Different conceptual models of frailty, including the phenotype model and deficit accumulation approach, emphasize its complex and heterogeneous nature (Rockwood and Mitnitski, 2007; Morley et al., 2013). Despite these differences, impaired physical performance—particularly in balance and mobility—remains a common feature across definitions. Clinically, balance dysfunction is often one of the earliest functional impairments observed in older adults with declining physiological reserve (Bellelli et al., 2024; Fried et al., 2001).

Maintaining postural stability requires continuous integration of sensory feedback, neuromuscular control, and cognitive processing. Disruption of any of these components can lead to instability, especially under dual-task or environmental perturbation conditions. Evidence from observational studies suggests that poor balance performance is a strong independent predictor of future falls in older populations, even after adjusting for comorbidities and general physical function (Horak, 2006; Beck Jepsen et al., 2022; Sherrington et al., 2019). Given that falls are a major cause of injury and loss of independence in older adults, balance impairment represents a clinically meaningful target for intervention.

Exercise-based interventions are widely recommended as a core strategy for maintaining functional ability in aging populations. International guidelines consistently support regular physical activity to improve strength, mobility, and reduce fall risk in older adults (Lee et al., 2017; Sherrington et al., 2020). However, adherence and safety concerns often limit participation in conventional high-intensity exercise programs among frail individuals, indicating the need for alternative approaches that are both tolerable and effective.

Mind–body exercise has gained increasing attention in this context. Practices such as Tai Chi, Qigong, Yoga, and Baduanjin combine slow and controlled movements with breathing regulation and attentional focus. This integrated approach allows simultaneous engagement of physical and cognitive systems while maintaining relatively low musculoskeletal load. Randomized controlled trials have reported that Tai Chi-based interventions may improve balance control and reduce fall risk in older adults, although findings vary depending on population characteristics and intervention design (Chen et al., 2023; Xu et al., 2023; Park et al., 2022).

Beyond individual trials, accumulating systematic evidence suggests that mind–body exercise may provide broader benefits for functional capacity in older adults with frailty or pre-frailty. However, most previous reviews have focused on general physical function or frailty status, while balance has often been treated as a secondary or composite outcome (Navarrete-Villanueva et al., 2021). As a result, the specific effects of mind–body exercise on different domains of balance control—such as static, dynamic, and overall balance—remain insufficiently clarified.

Another important gap concerns variability in response according to frailty stage. It is plausible that individuals in the pre-frail stage, who retain greater physiological reserve, may respond differently to exercise interventions compared with those in more advanced frailty states. However, current evidence is inconsistent, and few studies have directly addressed this issue using stratified analyses.

In addition, exercise prescription parameters vary substantially across studies. Frequency, session duration, and total intervention length differ widely, and there is limited consensus regarding optimal dosage for achieving balance improvements in frail populations. This heterogeneity limits the translation of research findings into practical rehabilitation guidelines.

Therefore, the present systematic review and meta-analysis was conducted to synthesize available evidence on the effects of mind–body exercise on overall, dynamic, and static balance in older adults with pre-frailty and frailty. Subgroup analyses were performed to explore whether frailty stage and exercise prescription characteristics influence intervention effects. This study aims to provide a more precise and clinically applicable evidence base for rehabilitation strategies targeting balance dysfunction in older adults.

## 2 Methods

### 2.1 Literature search strategy

A systematic search was conducted across PubMed, Web of Science, Cochrane Library, Embase, CNKI, Wanfang, and VIP databases from their inception to March 2026.

The search combined MeSH terms and free-text words, focusing on three categories (Rockwood and Mitnitski, 2007): mind-body exercise (e.g., “Tai Chi,” “Yoga,” “Qigong,” “Baduanjin”) (Morley et al., 2013); frailty (e.g., “frailty,” “frail elderly,” “pre-frail”) (Walston et al., 2006); balance function (e.g., “balance,” “postural control,” “falls”). Boolean operators were used to combine search terms.

### 2.2 Inclusion and exclusion criteria

#### 2.2.1 Inclusion criteria

Based on the PICO framework, studies were included if they met the following criteria (Rockwood and Mitnitski, 2007): Population (P): older adults aged ≥60 years who were frail, pre-frail or having clinically relevant physical vulnerability associated with frailty progression (Morley et al., 2013); Intervention (I): mind-body exercise including Tai Chi, Yoga, Baduanjin, etc., (Walston et al., 2006); Comparison (C): usual care, health education, or no intervention (Bellelli et al., 2024); Outcome (O): at least one objective balance measure, including the Berg Balance Scale (BBS) or Timed Up and Go (TUG) test (Fried et al., 2001); Study design: randomized controlled trials (RCTs).

#### 2.2.2 Exclusion criteria

1. Non-randomized studies (e.g., observational studies, reviews) (Morley et al., 2013); Studies not clearly involving frail or pre-frail populations, or without extractable subgroup data (Walston et al., 2006); Multi-component interventions without separate effect of mind-body exercise (Bellelli et al., 2024); Studies reporting only subjective outcomes (e.g., fear of falling) without objective balance measures (Fried et al., 2001); Incomplete or non-extractable data (Horak, 2006); Duplicate publications (preference given to the most complete dataset).

### 2.3 Outcome classification

Balance outcomes were classified into three functional domains according to their predominant assessment characteristics.

1. Overall balance: measures providing global balance performance, including BBS and Performance-Oriented Mobility Assessment (POMA);
2. Dynamic balance: measures involving movement-related postural control, transitional mobility, gait, and turning ability, including TUG, Five Times Sit-to-Stand Test (FTSST), Functional Reach Test (FRT), gait speed, and related mobility assessments;
3. Static balance: measures primarily reflecting postural stability during maintained positions or controlled weight shifting, including One-Leg Standing test (OLS)

This classification was prespecified before quantitative synthesis. Because several instruments assess overlapping constructs, classification was based on their predominant functional domain rather than representing isolated physiological mechanisms.

### 2.4 Study selection and data extraction

Articles were imported into EndNote for management. Two reviewers independently screened title and abstract to identify potentially eligible studies, followed by independent full-text assessment according to the predefined inclusion and exclusion criteria. Any disagreements regarding study eligibility were resolved through discussion between the two reviewers, and a third reviewer was consulted when consensus could not be reached. The study selection process was presented using a PRISMA flow diagram (Page et al., 2021).

Data extraction was independently performed by two reviewers using a standardized data extraction form. Extracted information included the first author, year of publication, study design, country of the corresponding author, country in which the study population was recruited, type of intervention, sample size, diagnostic criteria for frailty, frailty status, and outcome measures. When multiple publications reported results from the same study, the report containing the most complete dataset was included in the quantitative synthesis, while the remaining publications were retained as supplementary references.

### 2.5 Risk of bias assessment

The methodological quality of the included randomized controlled trials was assessed using the Cochrane Risk of Bias 2 (RoB 2) tool (Sterne et al., 2019). This instrument comprises 12 signaling questions, each of which is rated as “Yes,” “No,” “Probably Yes/No,” or “No Information,” and evaluates five domains: the randomization process, deviations from intended interventions, missing outcome data, measurement of the outcome, and selection of the reported result. Based on the study design and reporting characteristics, each domain was judged as having a “low risk of bias,” “some concerns,” or a “high risk of bias.” An overall risk-of-bias judgment was then assigned by integrating the assessments across all five domains.

To ensure the accuracy and consistency of the evaluations, two reviewers independently assessed the risk of bias of all included randomized controlled trials using the RoB two tool. The results of the risk-of-bias assessment were presented as risk-of-bias summary figures.

### 2.6 Effect size calculation and directionality

For continuous outcomes, Hedges’ g was used to correct for small-sample bias, and SMD with 95% CI were calculated. Effect size interpretation followed Cohen’s convention: small (0.2), medium (0.5), large (0.8).

Effect direction was standardized: positive SMD indicated improvement for overall and static balance while negative SMD indicated improvement for dynamic balance.

### 2.7 Statistical analysis

Meta-analysis was conducted using a random-effects model. Heterogeneity was quantified using I^2^ statistics. Subgroup analyses explored sources of heterogeneity and predefined moderators (Rockwood and Mitnitski, 2007): frailty stage (pre-frail vs. frail) (Morley et al., 2013); intervention frequency (moderate-to-low: 2–4 sessions/week vs. high: ≥5 sessions/week) (Walston et al., 2006); session duration (short: <30 min, medium: 31–60 min, long: >60 min) (Bellelli et al., 2024); intervention period (short: <12 weeks, medium: 13–24 weeks, long: >24 weeks).

Analyses were conducted using Review Manager 5.4, with a two-sided significance level of P < 0.05.

When multiple intervention arms from the same study shared a common control group, the shared control group was adjusted to avoid double counting. Specifically, the number of participants in the control group was divided proportionally among the comparisons involving different intervention arms. This approach was used to prevent artificial inflation of sample size and preserve the independence of effect estimates in the meta-analysis, following recommendations from the Cochrane Handbook for Systematic Reviews of Interventions.

Meta-regression was not performed because the number of studies available for each outcome was insufficient to provide reliable estimates. Instead, clinically prespecified subgroup analyses were conducted to explore potential sources of heterogeneity.

### 2.8 Sensitivity analysis

To assess the robustness of the pooled results and to explore potential sources of heterogeneity, a leave-one-out sensitivity analysis was performed. In this procedure, each included study was sequentially excluded, and the remaining randomized controlled trials (RCTs) were reanalyzed using a random-effects model. The pooled SMD and 95% CI were recalculated after each exclusion and compared with the corresponding estimates derived from the full dataset. Two aspects were examined in particular (Rockwood and Mitnitski, 2007): whether the direction of the pooled effect estimate was reversed or its statistical significance changed after exclusion of an individual study; and (Morley et al., 2013) whether the heterogeneity statistic (I^2^) showed a substantial reduction. A study was considered to have a substantial influence on the overall pooled result if, after its removal, the point estimate of the pooled effect fell outside the 95% CI of the full-model estimate or if the I^2^ value decreased by more than 10 percentage points. The sensitivity analysis was conducted manually using Review Manager version 5.4.

### 2.9 PROSPERO registration

This systematic review was registered in PROSPERO (CRD420261406159).

## 3 Results

### 3.1 Study selection and characteristics

**FIGURE 1:**
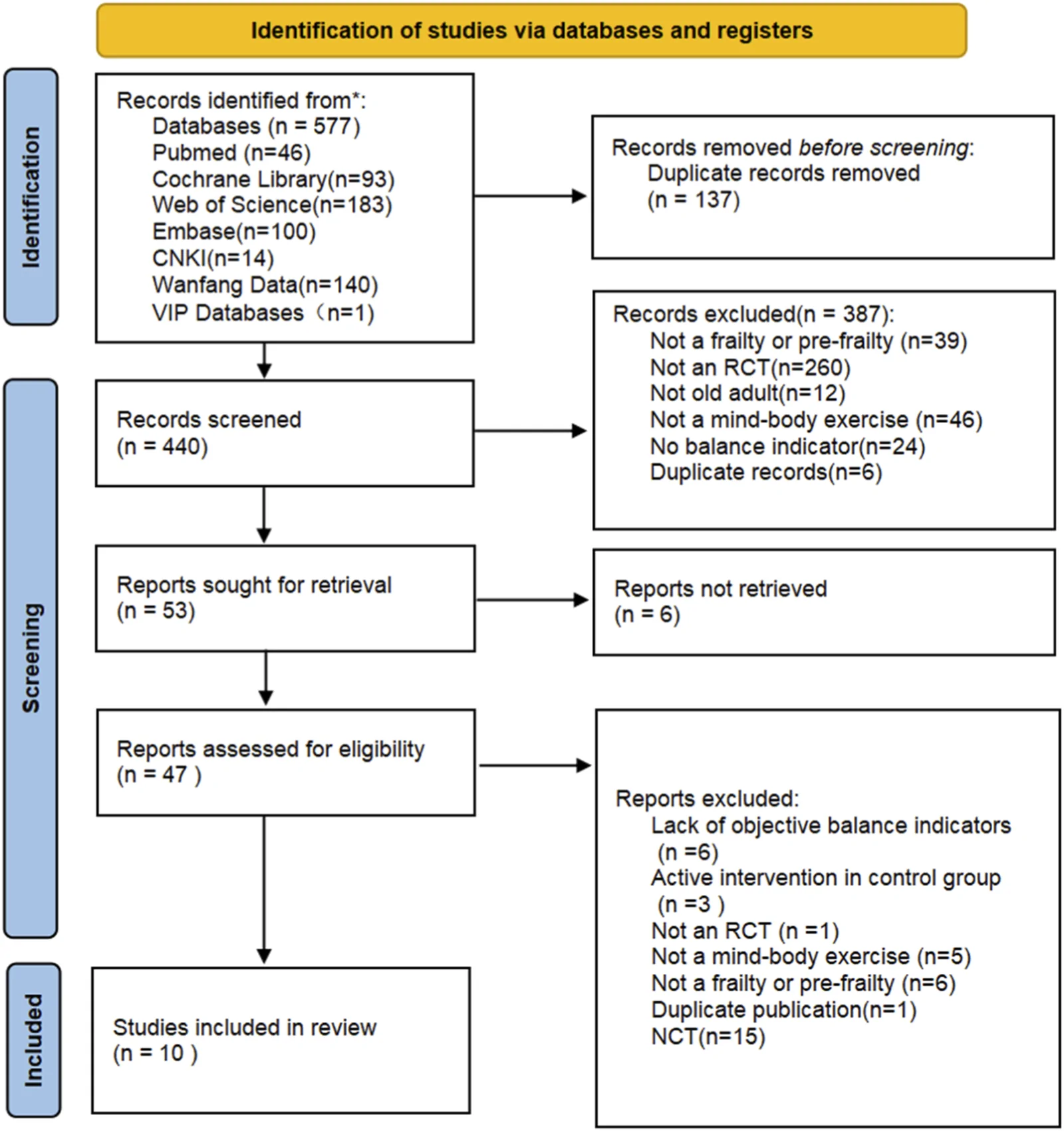
PRISMA flow diagram for searching of the included study.

A total of 577 articles were initially retrieved. After deduplication and screening using EndNote and full-text review, 10 studies were included, 5 English (Hass et al., 2004; Faber et al., 2006; Manor et al., 2014; Saravanakumar et al., 2014; Wolf et al., 2006), 5 Chinese (Gao, 2020; Ge et al., 2020; Qiu, 2023; Wu, 2022; Zhu, 2016). The included studies were published between 2014 and 2023, conducted in China, the United States, Australia, and other countries. Tai Chi was the most common intervention, followed by Baduanjin and Yoga. The PRISMA flow diagram is shown in Figure 1 and study characteristics are summarized in Table 1.

**TABLE 1:** The Characteristics of included studies (1 = TUG, 2 = FTSST, 3 = OLS, 4 = SPPB,5 = Gait speed, 7 = FRT, 8 = COP, 9 = POMA, 10 = BBS).

Study ID	Country	Sample size (I/C)	Age (Mean ± SD,I/C)	Frailty assessment tool	Frailty stage	Intervention	Intervention duration	Control	Balance outcome measures
Gao_2020	China	34/34	79.79 ± 4.18/78.88 ± 4.66	Fried Frailty phenotype	Pre-frail	Baduanjin	12 weeks	Usual activity + weekly health education	1,2,3
Manor_2014	United States	29/28	87 ± 5/86 ± 6	Fried Frailty phenotype	Frail/Pre-frail	Tai chi	12 weeks	Health education	1,4,5,10
Qiu_2023	China	21/20	73.53 ± 6.04/70.90 ± 5.11	Tilburg frailty indicator (Chinese version)	Frail	Yoga	12 weeks	Free activity	1,2
Wu_2022	China	54/48	66.9 ± 4.54/67.64 ± 5.49	Edmonton Frail scale	Frail	Baduanjin	24 weeks	Dietary guidance	1,3,7,9
Zhu_2016_1	China	28/27	64 ± 3/64 ± 4	Asian sarcopenia criteria	Pre-frail	Tai chi	72 weeks	Sarcopenia education	1,2,3,5
Zhu_2016_2	China	24/27	88.8 ± 3.7/87.5 ± 3.0	Frailty status evaluation	Frail	Tai chi	8 weeks	Sarcopenia education	1,2,5
Ge_2020	China	32/30	70.16 ± 5.40/72.91 ± 6.61	Fried frailty phenotype	Pre-frail	Tai chi	8 weeks	Blank control	3,7
Wolf_2006	United States	98/94	80.8 ± 5.9/81.0 ± 6.4	Not specified (described as transitionally frail)	Frail	Tai chi	48 weeks	Health education	5,7
Saravanakumar_2014_1	Australia	8/10	81.1 ± 8.0/85.4 ± 9.1	Not specified (described as frail)	Frail	Tai chi	14 weeks	Usual care	10
Saravanakumar_2014_2	Australia	8/10	84.9 ± 6.7/85.4 ± 9.1	Not specified (described as frail)	Frail	Yoga	48 weeks	Usual care	10
Hass_2004	United States	14/14	79.5 ± 6.0/79.7 ± 5.6	Speechley and tinetti criteria	Frail/Pre-frail	Tai chi	48 weeks	Health education	8
Faber_2006	Netherlands	70/84	84.4 ± 6.4/84.9 ± 5.9	Fried Frailty phenotype	Frail/Pre-frail	Tai chi-based exercise	20 weeks	Blank control	9

### 3.2 Risk of bias assessment

**FIGURE 2:**
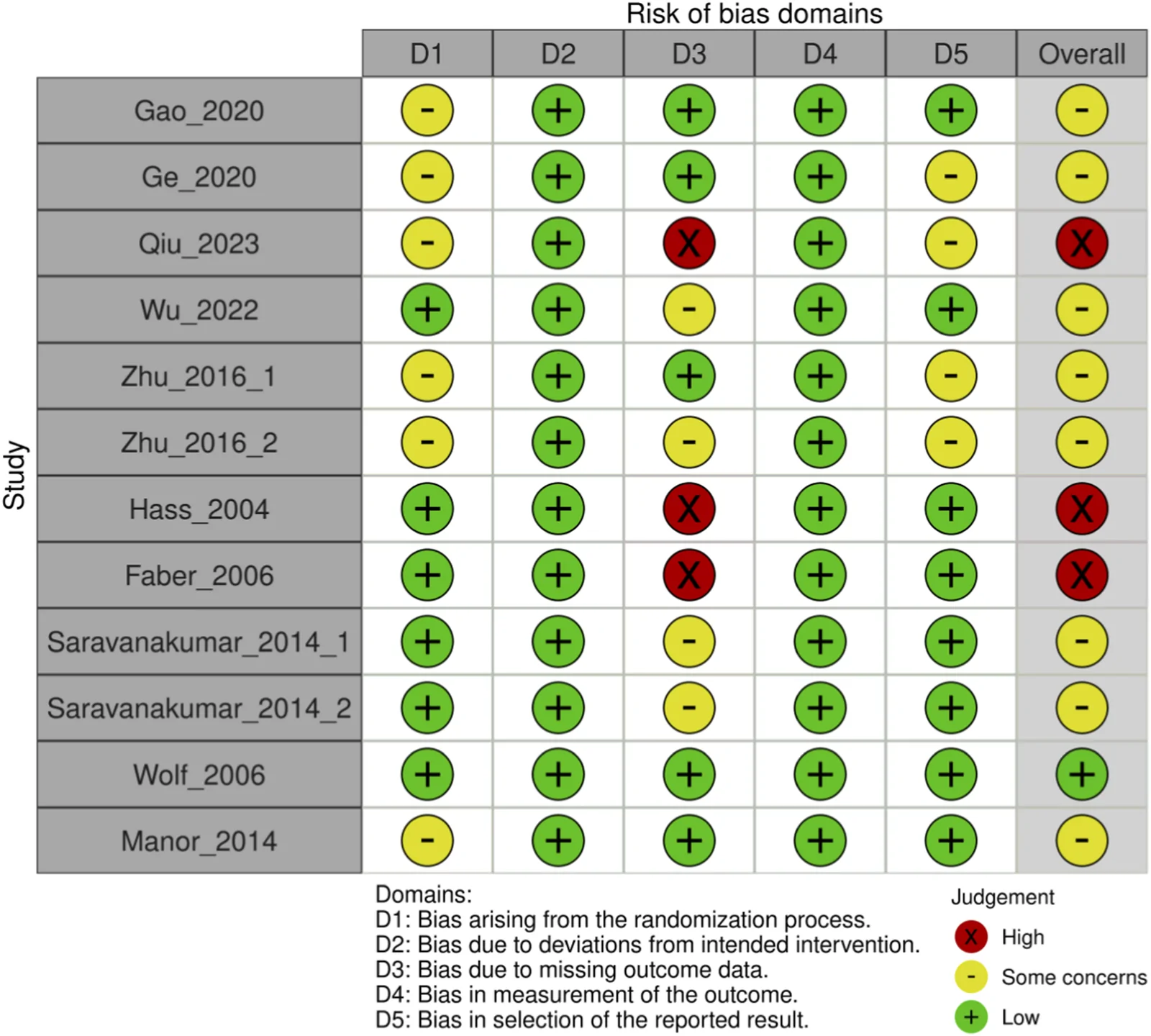
Risk of bias graph of each included study.

**FIGURE 3:**
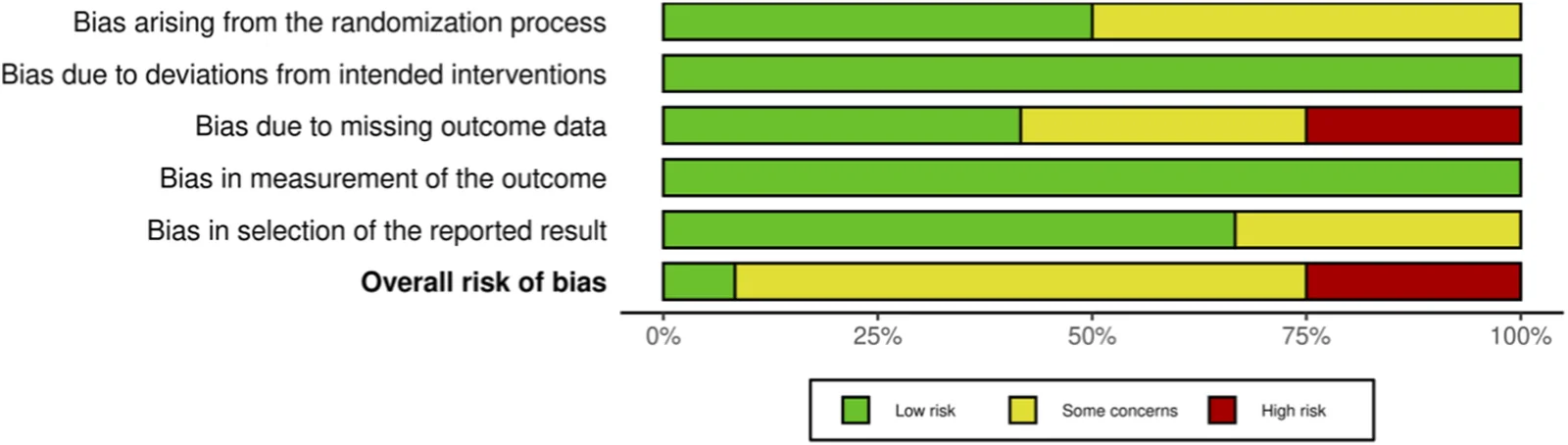
Proportional distribution of risk of bias across domains.

Overall methodological quality was moderate. Most studies reported adequate randomization, but blinding was generally not feasible. The majority were rated as having some concerns (Figures 2, 3).

### 3.3 Overall effects of mind-body exercise on balance

#### 3.3.1 Overall balance function

**FIGURE 4:**
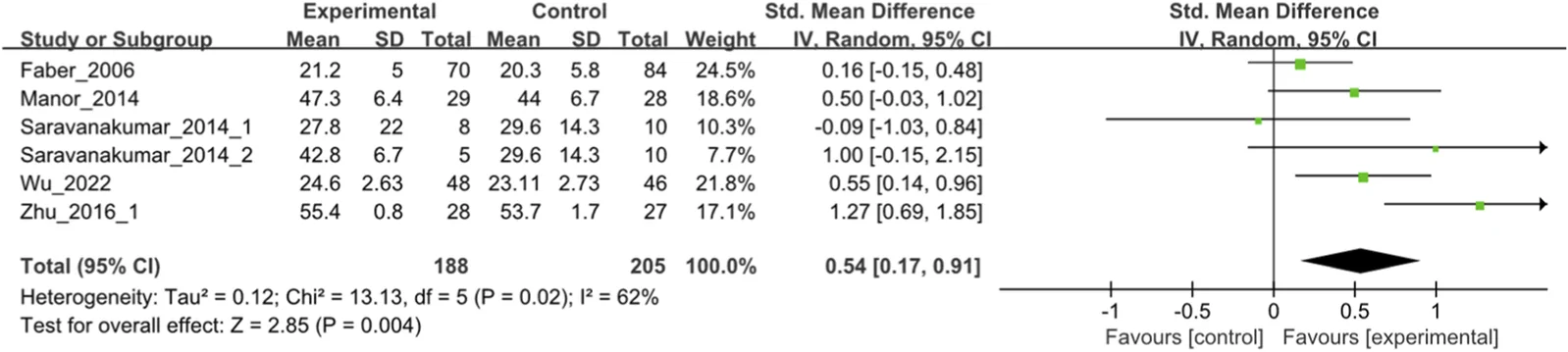
Forest plot of the effect of mind–body exercise on overall balance function in pre-frail and frail older adults.

Six studies reported overall balance outcomes, mainly using BBS and POMA, with BBS as the primary outcome. Mind–body exercise improved overall balance (SMD = 0.54, 95% CI [0.17–0.91], P = 0.004) (Figure 4), with moderate heterogeneity. In addition, the BBS has a maximum score of 56 points, and in older adults, a change of 4–7 points is generally regarded as a genuine and clinically meaningful improvement. Therefore, the magnitude of the effect observed in the present study is of potential clinical relevance.

#### 3.3.2 Dynamic balance function

**FIGURE 5:**
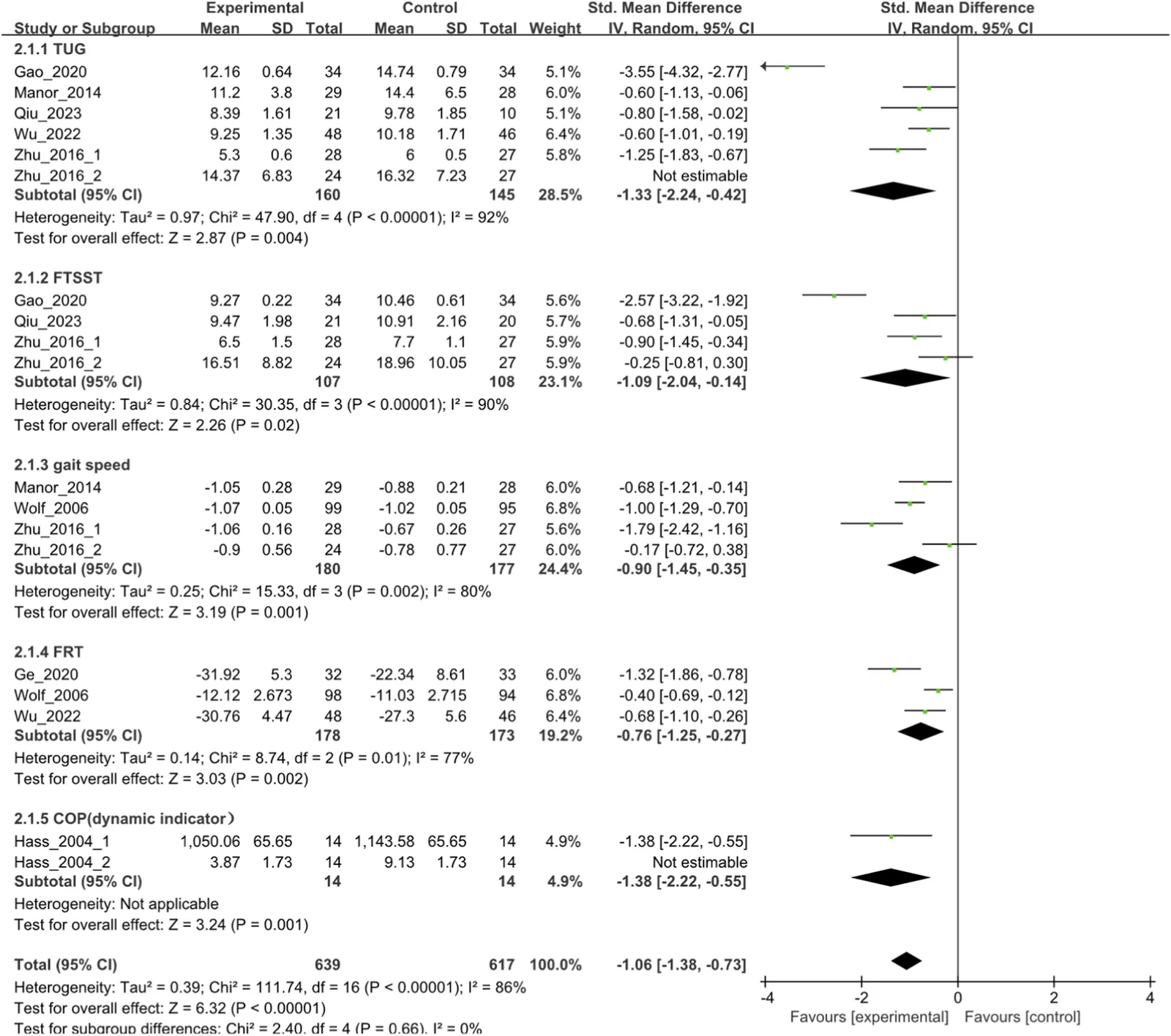
Forest plot of the effect of mind–body exercise on dynamic balance function in pre-frail and frail older adults.

Dynamic balance was primarily assessed using the TUG test, supplemented with FTSST, gait speed, FRT, and center of pressure (COP). The pooled SMD was −1.08 (95% CI [-1.41, −0.76]), demonstrating a large effect of mind-body exercise on dynamic balance (P < 0.00001), with high heterogeneity (I^2^ = 86%, P < 0.00001), this finding indicates substantial between-study variability, which may be attributable to differences in intervention protocols, frailty status, or the specific outcome measures used (Figure 5).

#### 3.3.3 Static balance function

**FIGURE 6:**
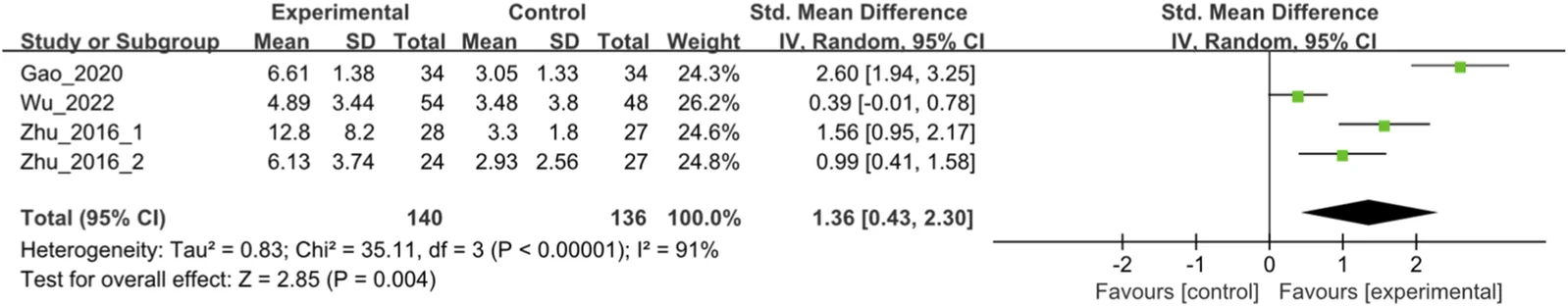
Forest plot ofthe effect of mind–body exercise on static balance function in pre-frail and frail older adults.

Four studies assessed static balance, primarily using OLS. The pooled SMD was 1.36 (95% CI [0.43, 2.30]), indicating a large and statistically significant improvement in static balance (P = 0.004), with very high heterogeneity (I^2^ = 91%, P < 0 0.00001), the cautious interpretation of the pooled effect size may be partly attributed to the small number of included studies and variations in measurement protocols (Figure 6).

### 3.4 Subgroup analysis

#### 3.4.1 Frailty stage

Subgroup analysis data for frailty stage were provided by 10 studies (Faber_2006 and Hass_2004 could not be included in this analysis due to mixed populations). The subgroup analysis showed that the pooled SMD for pre-frail individuals was 1.62 (95% CI [0.53, 2.72]), indicating a large and significant effect, albeit with high heterogeneity (I^2^ = 93%, P < 0.00001), suggesting considerable differences in intervention characteristics among the pre-frail studies. The pooled SMD for frail individuals was 0.45 (95% CI [0.25, 0.65]), indicating a small-to-moderate effect, which was stable with low heterogeneity (I^2^ = 0%, P = 0.55).

**FIGURE 7:**
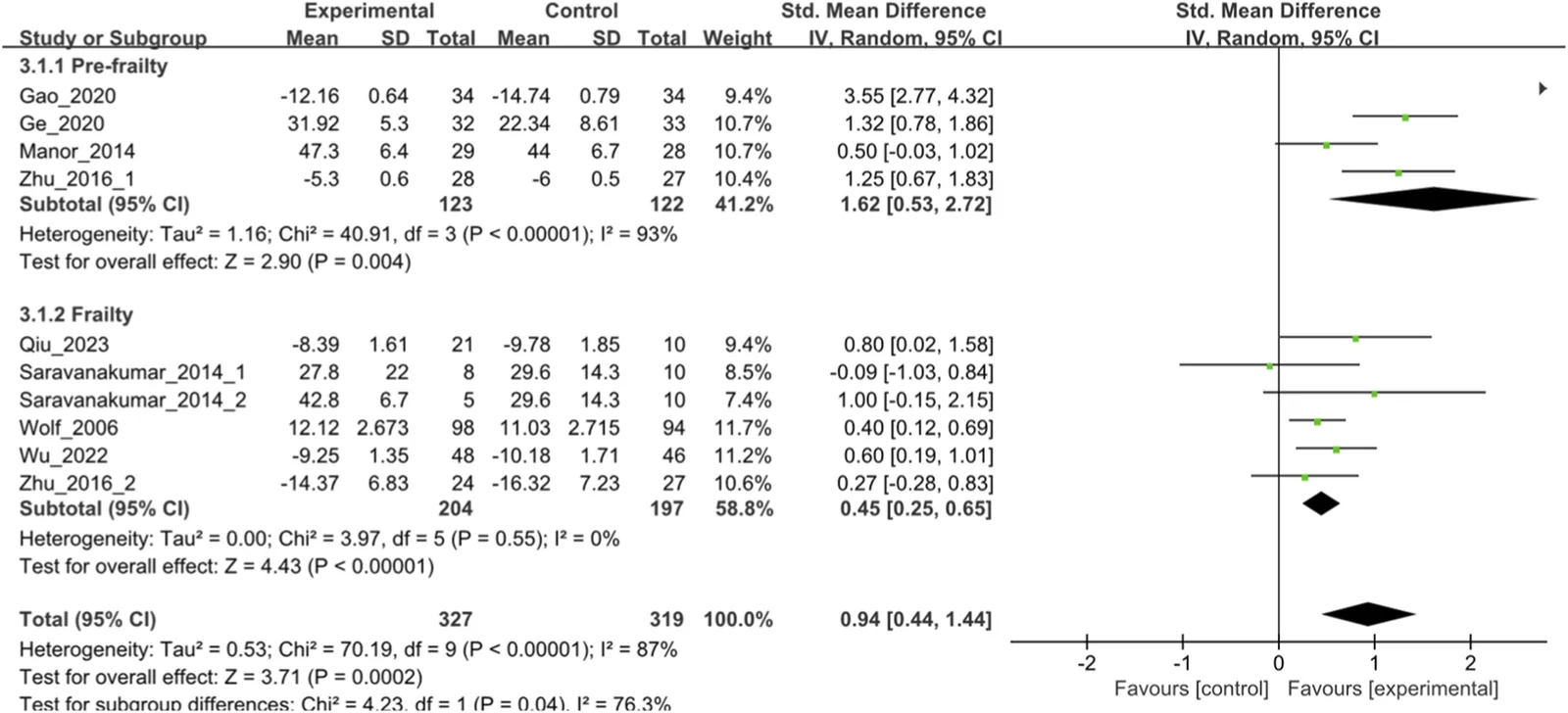
Forest plot of subgroup analysis for the effect of different frailty stages on balance function intervention outcomes.

The subgroup difference test was statistically significant (Chi^2^ = 4.23, df = 1, P = 0.04), confirming that pre-frail older adults achieved significantly greater balance improvement from mind-body exercise interventions than frail older adults. Based on relevant medical knowledge, the potential reason for this finding is that pre-frail older adults retain higher physiological functional reserves, which may lead to stronger responses to exercise interventions (Figure 7).

#### 3.4.2 Intervention frequency

Data from 12 comparison groups were included in the subgroup analysis by intervention frequency. The subgroup analysis showed that the pooled SMD for low-to-moderate frequency (2–4 sessions/week) was 0.62 (95% CI [0.33, 0.91]), indicating a moderate and significant effect (P < 0.0001), with moderate heterogeneity (I^2^ = 62%). This regimen appears stable and clinically feasible. For high frequency (≥5 sessions/week), the pooled SMD was 1.67 (95% CI [−0.06, 3.40]), indicating a large effect size that did not reach statistical significance (P = 0.06), with very high heterogeneity (I^2^ = 96%, P < 0.00001).

**FIGURE 8:**
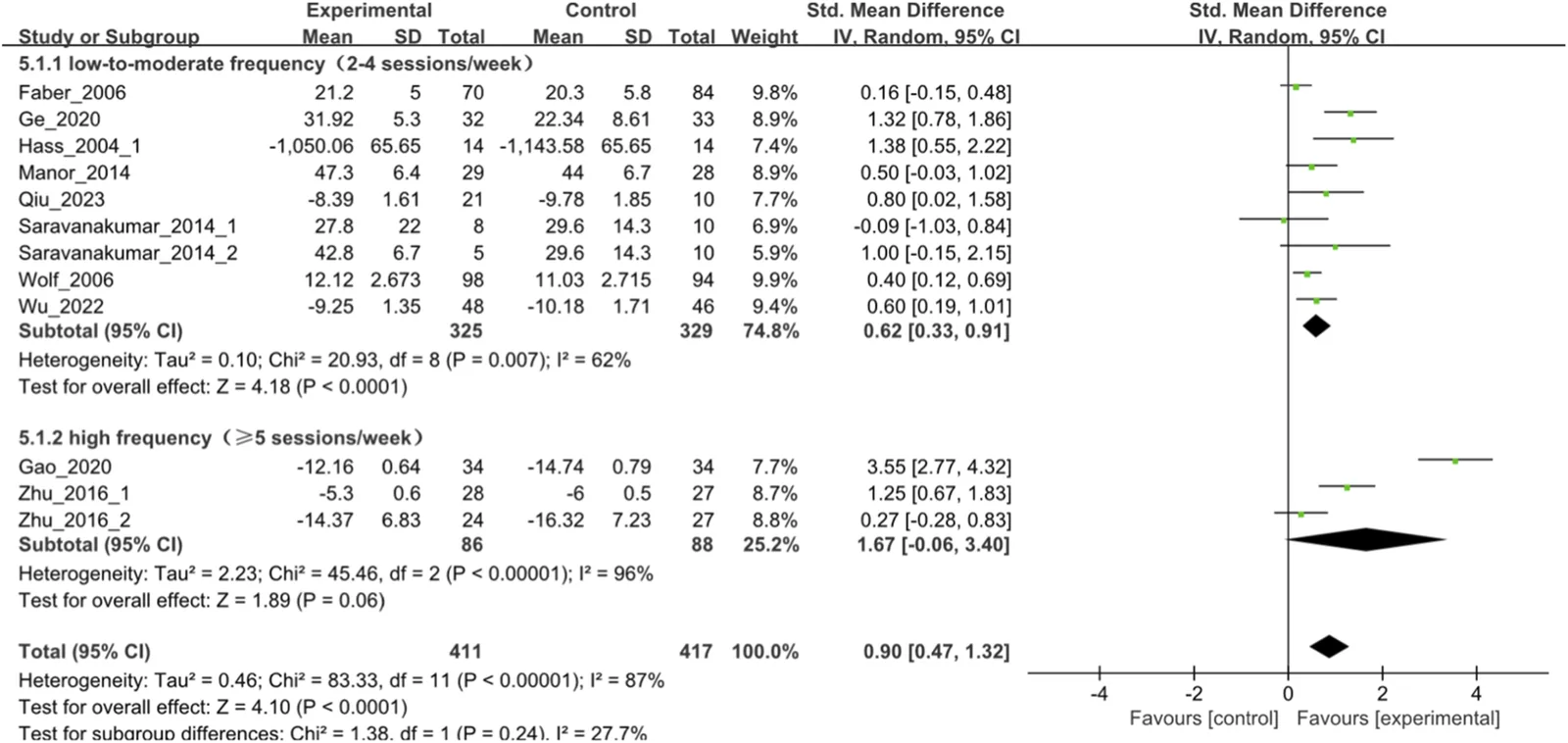
Forest plot of subgroup analysis for the effect of intervention frequency on balance ability outcomes in pre-frail or frail older adults.

The subgroup difference was not statistically significant (Chi^2^ = 1.38, df = 1, P = 0.24), primarily due to the insufficient precision of the high-frequency estimate—i.e., an excessively wide confidence interval. From a practical perspective, low-to-moderate frequency interventions are more reliable and generalizable, whereas evidence for high-frequency regimens is limited by small sample sizes and excessive between-study variability (Figure 8).

#### 3.4.3 Session duration

**FIGURE 9:**
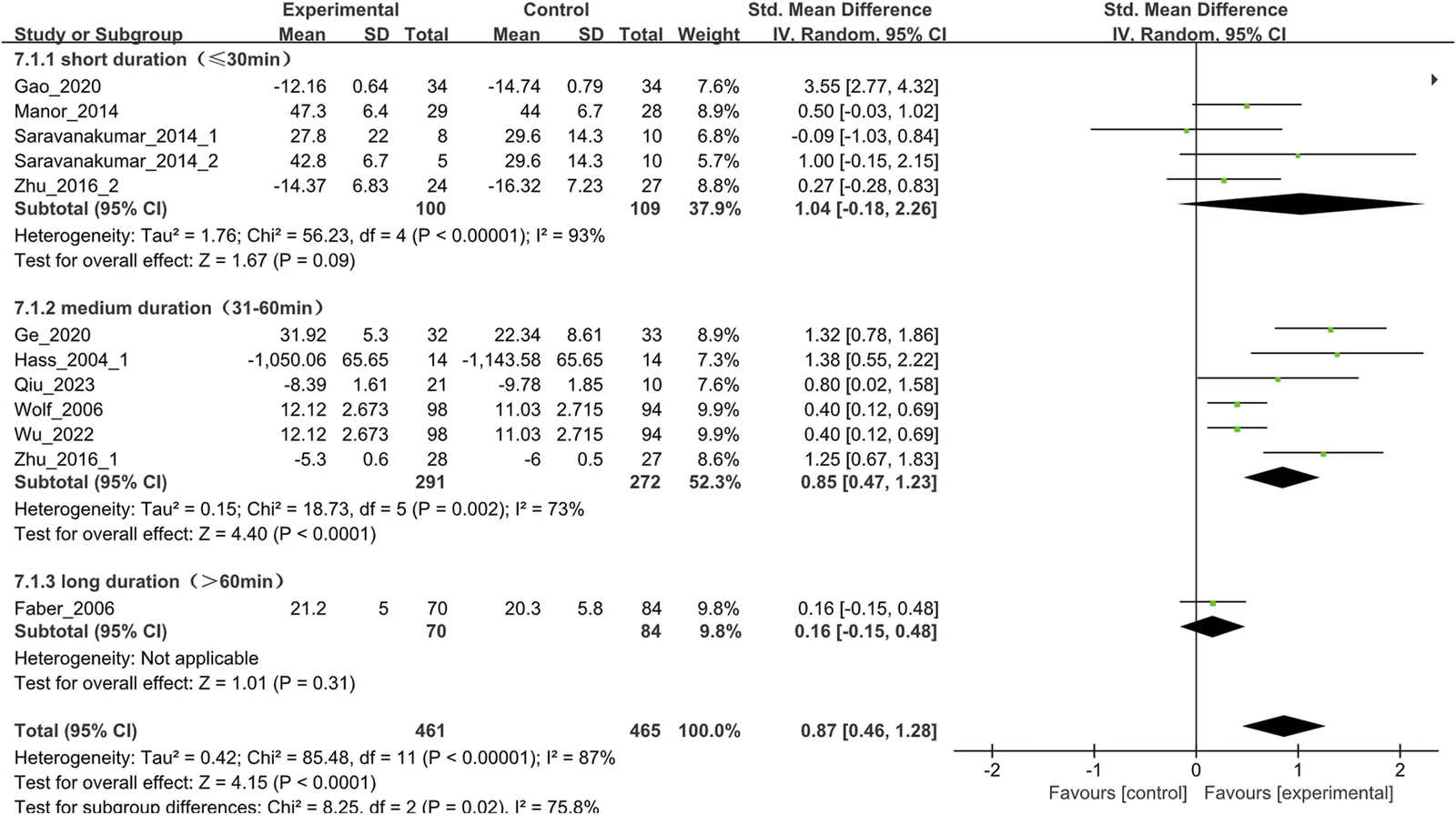
Forest plot of subgroup analysis for the effect of different single-session intervention duration on balance function in pre-frail and frail older adults.

Subgroup analysis showed that for medium duration (31–60 min), the pooled SMD was 0.85 (95% CI [0.47, 1.23]), indicating a moderate-to-large and significant effect (P < 0.0001), with moderate heterogeneity (I^2^ = 73%). This duration yielded the most robust intervention effects. For short duration (<30 min), the pooled SMD was 1.04 (95% CI [−0.18, 2.26]), indicating a large effect size that did not reach statistical significance (P = 0.09), with high heterogeneity (I^2^ = 93%) and insufficient precision to draw definitive conclusions. For long duration (>60 min), insufficient data were available for meta-analysis (Figure 9).

These findings suggest that 31–60 min is associated with relatively more consistent improvements in balance function among older adults. A duration of less than 30 min may be insufficient to induce meaningful neuromuscular adaptations, whereas longer durations may increase the execution burden on older adults without necessarily providing additional benefits.

#### 3.4.4 Intervention period

**FIGURE 10:**
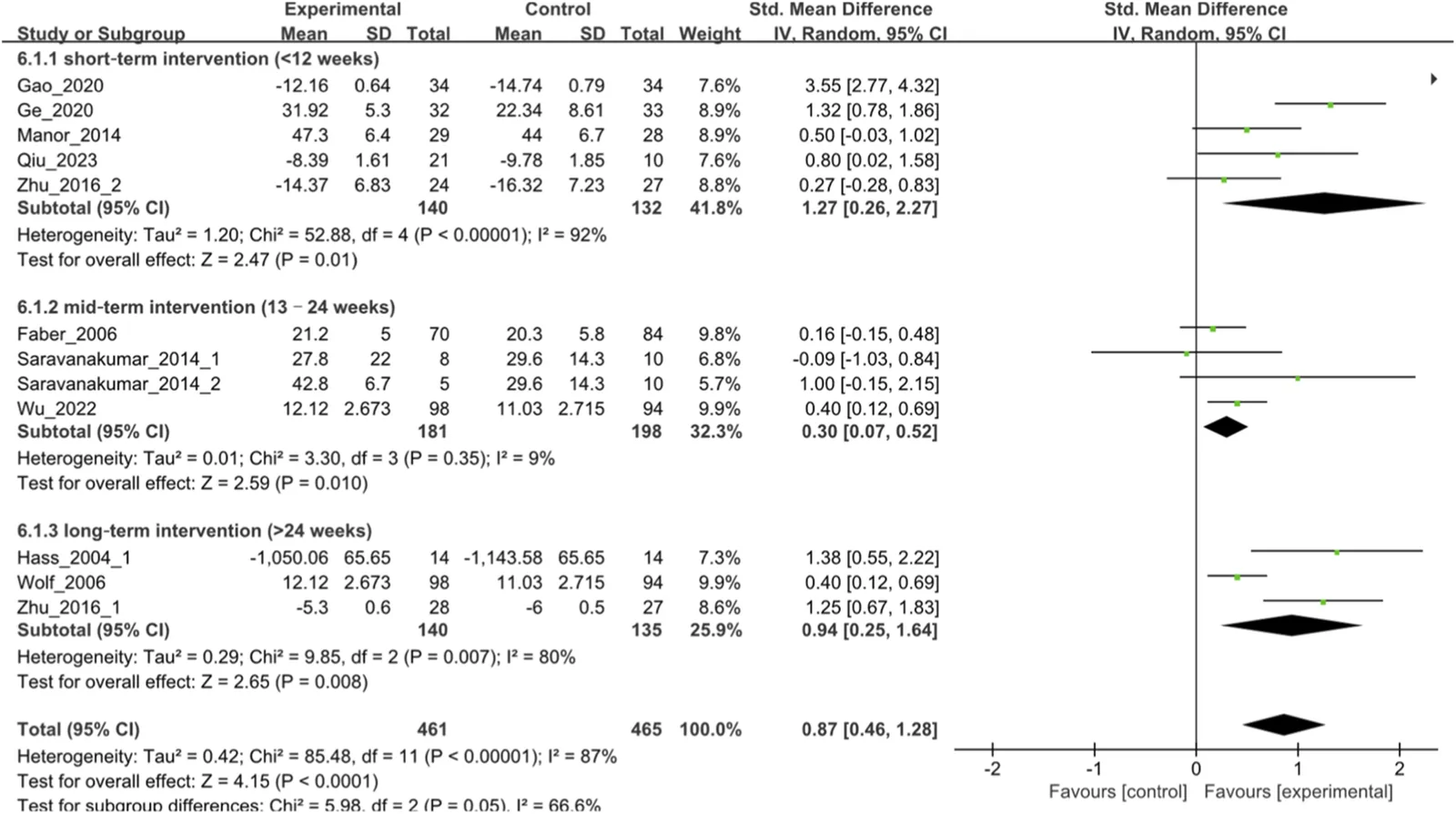
Forest plot of subgroup analysis for the effect of different intervention duration on balance function in pre-frail and frail older adults.

Subgroup analysis results showed that for mid-term intervention (13–24 weeks), the pooled SMD was 0.30 (95% CI [0.07, 0.52]), indicating a small-to-moderate effect size but the most stable estimate, with very low heterogeneity (I^2^ = 9%, P = 0.010). This regimen is the most robust, demonstrating high consistency across studies. For long-term intervention (>24 weeks), the pooled SMD was 0.94 (95% CI [0.25, 1.64]), indicating a large effect size but with high heterogeneity (I^2^ = 80%, P = 0.008), potentially attributable to differences in intervention content or participant characteristics across longer-duration studies. For short-term intervention (<12 weeks), the pooled SMD was 1.27 (95% CI [0.26, 2.27]), indicating a large effect size but with insufficient precision and very high heterogeneity (I^2^ = 92%, P = 0.01). The subgroup difference approached statistical significance (Chi^2^ = 5.98, df = 2, P = 0.05) (Figure 10), suggesting that intervention duration indeed moderates the intervention effect. Mid-term intervention achieves the best balance between effect stability and practical feasibility.

### 3.5 Sensitivity analysis

Leave-one-out sensitivity analyses demonstrated that the pooled estimates for comprehensive, dynamic, and static balance were generally robust.

For comprehensive balance, omission of any single study did not materially alter the pooled effect size or its statistical significance. Notably, exclusion of Zhu_2016_1 reduced heterogeneity from 62% to 13% while the pooled effect remained significant (SMD = 0.36, 95% CI 0.17–0.55), indicating that this study was a major contributor to heterogeneity but not to the direction of the overall effect.

For static balance, the pooled effect remained significant after sequential exclusion of each study (pooled SMD range: 0.97–1.67). Heterogeneity remained high (I^2^ range: 81%–94%), suggesting that between-study variability was attributable to distributed methodological and clinical differences rather than a single outlying study.

For dynamic balance, exclusion of Gao_2020 substantially reduced heterogeneity within the TUG subgroup (from 91% to 34%), yet the pooled estimate remained statistically significant. Although omission of several studies led to loss of statistical significance in some secondary dynamic outcomes (e.g., FTSST, gait speed, and FRT), the direction and magnitude of effect were largely unchanged. Collectively, these findings support the robustness of the overall conclusion that mind-body exercise improves dynamic balance.

## 4 Discussion

The present review indicates that mind–body exercise can improve several aspects of balance performance in older adults with pre-frailty or frailty. Improvements were observed in overall, dynamic, and static balance outcomes. Given the strong association between impaired balance and adverse events such as falls, disability, and functional decline, these findings may have important implications for geriatric rehabilitation. However, the interpretation of these findings is constrained by considerable between-study heterogeneity and the limited number of studies available for certain outcomes, necessitating a cautious approach to clinical translation.

Impaired balance is now considered a characteristic feature of frailty rather than a simple consequence of advancing age. Frailty is characterized by diminished physiological reserve and reduced resilience across multiple biological systems, leading to increased vulnerability to adverse health outcomes, including falls, disability, hospitalization, and mortality (Clegg et al., 2013; Hoogendijk et al., 2019; Dent et al., 2026). Successful postural control depends on coordinated interactions among musculoskeletal, sensory, vestibular, neural, and cognitive systems. Consequently, age-related deterioration across these domains may contribute to impaired postural control among frail individuals. From a clinical perspective, interventions capable of simultaneously targeting multiple physiological systems may therefore be particularly beneficial.

The overall findings of the present study are broadly consistent with previous evidence supporting exercise as a core component of frailty management. Several systematic reviews have reported that exercise interventions can improve physical performance, mobility, and frailty-related outcomes in older adults (Puts et al., 2017; de Labra et al., 2015; Apóstolo et al., 2018). However, relatively few reviews have specifically focused on balance function despite its strong association with fall risk and functional independence. Therefore, our findings extend the current evidence base by suggesting that mind–body exercise may provide clinically meaningful improvements across multiple dimensions of balance performance.

Among the balance domains examined, the greatest effect was observed for dynamic balance. Dynamic balance underpins many daily activities, including walking, directional changes, stair climbing, and obstacle negotiation, and impairments in dynamic balance have been consistently associated with an elevated risk of falls among older adults (Muir et al., 2010; Kojima, 2015). Furthermore, emerging evidence suggests that mobility and balance are closely linked to broader neurological and cognitive processes. For example, gait variability has been proposed as a marker of neurological vulnerability and future adverse outcomes, while the concept of Motoric Cognitive Risk Syndrome highlights the close relationship between mobility impairment and cognitive decline (Hausdorff et al., 2001; Montero-Odasso et al., 2020; Xiang et al., 2022). Improvements in dynamic balance may therefore contribute not only to mobility but also to broader aspects of functional independence. By contrast, static balance, although demonstrating the largest pooled effect size (SMD = 1.36, 95% CI 0.43–2.30), was assessed in only four studies with very high heterogeneity (I^2^ = 91%), which substantially limits the certainty of this estimate. The GRADE assessment indicated that the certainty of evidence for static balance was very low, primarily due to serious inconsistency and imprecision (Supplementary Table S2). Therefore, the observed effect on static balance should be interpreted with particular caution, and the finding requires confirmation in future studies using standardized measurement protocols.

The beneficial effects observed in this review are likely multifactorial. Although differing in form, Tai Chi, Baduanjin, and yoga all incorporate controlled movements, postural adjustments, weight transfer, breathing coordination, and focused attention. These elements may collectively stimulate sensory integration, neuromuscular coordination, proprioceptive function, and postural control. Previous research has suggested that attentional resources and executive function play important roles in balance regulation among older adults (Woollacott and Shumway-Cook, 2002). In addition, some studies have reported improvements in cognitive performance following mind–body exercise interventions (Taylor-Piliae et al., 2006; Yu et al., 2022). However, because cognitive outcomes were not consistently reported across the studies included in this review, it remains unclear whether cognitive improvements directly contributed to the observed balance gains. However, the available evidence remains insufficient to establish a direct causal link between cognitive changes and balance improvement.

Subgroup analyses suggested that participants with pre-frailty may experience greater benefits than those with established frailty. Individuals in the pre-frail stage generally retain more physiological reserve and functional capacity than those with established frailty, which may partly explain the larger treatment effects observed, allowing more favorable responses to exercise interventions. Nevertheless, the current evidence does not allow direct examination of this mechanism. Alternative explanations, including differences in baseline functional status, intervention adherence, comorbidity burden, or study characteristics, cannot be excluded. Taken together, these findings reinforce the value of initiating exercise interventions before frailty becomes fully established. The GRADE assessment indicated moderate certainty for the finding of greater improvement in pre-frail compared with frail participants, though this is based on indirect subgroup comparisons rather than direct head-to-head trials (Supplementary Table S2).

The subgroup analyses provide preliminary insight into exercise prescription, although the results should be interpreted with caution. Although subgroup analyses indicated that interventions delivered two to four times per week, lasting approximately 31–60 min per session and maintained for at least 13 weeks tended to be associated with larger effect sizes, these observations were derived from indirect comparisons across studies rather than formal dose–response analyses. As a result, the available evidence remains insufficient to establish definitive recommendations regarding optimal exercise frequency, session duration, or intervention length. Additional trials specifically designed to evaluate dose–response relationships are needed before firm recommendations can be made.

Regarding intervention types, Tai Chi was the most frequently investigated modality, accounting for the majority of included studies, whereas Baduanjin and yoga were each examined in only a limited number of trials. Owing to the small number of studies available for each modality, formal modality-specific subgroup analyses were not feasible. Consequently, whether one form of mind–body exercise is superior to another remains unclear. It is possible that different modalities may produce distinct physiological adaptations due to differences in movement characteristics, cognitive engagement, training intensity, and cultural context, but this hypothesis requires direct comparative trials to be adequately tested.

Several methodological factors may have contributed to the heterogeneity observed across analyses. First, different studies employed a variety of balance assessment tools, including the BBS, TUG, FRT, and OLS. Because these instruments assess distinct aspects of balance performance, differences in responsiveness may have influenced pooled estimates. Second, comparator conditions varied substantially across studies, ranging from usual care and health education to active exercise controls. Such differences may have affected estimated treatment effects. Third, frailty definitions were not uniform across the included studies, with some adopting the Fried phenotype, others using the Edmonton Frail Scale or Tilburg Frailty Indicator, and one study employing sarcopenia-related criteria. This variation may have introduced clinical heterogeneity because different frailty assessment approaches capture partially overlapping but non-identical constructs of physiological vulnerability. Fourth, differences in intervention protocols, including session frequency, duration, and total program length, may have contributed to between-study variability. The substantial heterogeneity observed in several pooled analyses suggests that these methodological and clinical differences were not fully explained by our prespecified subgroup analyses.

It is also noteworthy that not all findings were uniformly positive. While significant improvements were observed in the primary analyses, some subgroup analyses failed to reach statistical significance. These non-significant findings may reflect limited statistical power, small sample sizes, methodological differences, or substantial between-study heterogeneity rather than a true absence of intervention effects. Such inconsistencies highlight the complexity of exercise responses in frail populations and reinforce the need for cautious interpretation of subgroup findings.

Sensitivity analyses supported the robustness of the primary findings. For overall balance, exclusion of Zhu et al. substantially reduced heterogeneity from 62% to 13% while preserving statistical significance, suggesting that this study contributed disproportionately to between-study variability but did not alter the overall direction of effect. Similarly, removal of Gao et al. markedly reduced heterogeneity within the TUG subgroup while the pooled effect remained significant, further supporting the stability of the overall findings. In contrast, heterogeneity in static balance remained consistently high despite sequential exclusion of individual studies, indicating that variability was likely attributable to distributed methodological and clinical differences rather than a single influential trial.

This review has several limitations that should be considered when interpreting the findings. The primary limitation is the considerable heterogeneity observed across several pooled analyses. While overall balance demonstrated moderate heterogeneity (I^2^ = 62%), heterogeneity was particularly high for dynamic balance (I^2^ = 86%) and static balance (I^2^ = 91%). Such variability likely reflects differences in participant characteristics, frailty definitions, intervention modalities, exercise dosage, outcome measures, and comparator conditions. Second, although all included studies were randomized controlled trials, methodological quality varied. In particular, blinding of participants and personnel was often difficult because of the nature of exercise interventions, potentially increasing the risk of performance bias. Third, Publication bias could not be fully evaluated for several outcomes because the number of included studies was limited, the relatively small number of studies available for some outcomes may have limited the power of these methods. Fourth, sensitivity analyses generally supported the robustness of the pooled findings, however, several outcomes remained influenced by individual studies, suggesting that the results should be interpreted cautiously. In addition, the scarcity of long-term follow-up data limits conclusions regarding the persistence of treatment benefits. Sixth, the generalizability of our findings may be constrained by the geographic distribution of the included studies, most of which were conducted in China, with fewer studies from the United States, Australia, and the Netherlands. Cultural and healthcare system differences may influence both intervention delivery and participant adherence, and therefore the results may not be directly transferable to other populations or settings. Finally, the GRADE assessment indicated that the certainty of evidence was moderate for overall balance, low for dynamic balance, and very low for static balance (Supplementary Table S2), primarily due to serious inconsistency, imprecision, and risk of bias. This underscores the need for additional high-quality trials to strengthen the evidence base.

Despite these limitations, the findings have important implications for clinical practice and public health. As global population aging accelerates, maintaining functional independence has become a major healthcare priority. Mind–body exercise possesses several characteristics that support implementation in both community and healthcare settings, including low cost, minimal equipment requirements, adaptability to different functional levels, and generally favorable safety profiles. These characteristics align closely with contemporary healthy aging frameworks proposed by the World Health Organization, which emphasize preservation of intrinsic capacity and functional ability throughout later life (Beard et al., 2015; World Health Organization, 2025). However, given the current limitations in evidence certainty and the exploratory nature of the dose-related findings, implementation decisions should be tailored to individual patient characteristics and resource availability, rather than applied as uniform prescriptive recommendations.

Future research should move beyond simply demonstrating efficacy and focus on identifying optimal intervention strategies. Direct head-to-head comparisons among Tai Chi, Baduanjin, and yoga are needed to determine modality-specific effects. Studies incorporating both cognitive and motor outcomes may help clarify the mechanisms underlying balance improvement. In addition, investigations examining dual-task performance, long-term maintenance of intervention effects, adherence, implementation feasibility, and cost-effectiveness would provide valuable information for clinical decision-making and large-scale community dissemination.

## 5 Conclusion

The available evidence suggests that mind–body exercise may improve balance performance in older adults with pre-frailty or frailty. Greater improvements were observed among individuals with pre-frailty than those with established frailty, although this finding should be interpreted cautiously because it was based on subgroup comparisons rather than direct comparisons between frailty stages. Exploratory subgroup analyses indicated that interventions with moderate frequency (2–4 sessions/week), approximately 31–60 min per session, and intervention periods of at least 13 weeks may be associated with more consistent improvements. However, these findings should be considered hypothesis-generating rather than definitive exercise prescriptions, and further dose–response randomized controlled trials are needed to establish optimal intervention parameters.
