# Supplementary material for: Effects of mind-body exercise on balance function in pre-frail and frail older adults: a systematic review and meta-analysis
Source: Front Aging. 2026 Sep 8;7:1915136. doi: 10.3389/fragi.2026.1915136 (PMC13595580; doi:10.3389/fragi.2026.1915136)
Supplement: Supplementary file 1 [file DataSheet1.pdf]

Supplementary Materials

Database	Search strategy	Search date
PubMed	(“Tai Ji”[Mesh] OR “Yoga”[Mesh] OR “Qigong”[Mesh] OR tai chi[Title/Abstract] OR yoga[Title/Abstract] OR qigong[Title/Abstract] OR baduanjin[Title/Abstract] OR yijinjing[Title/Abstract] OR “mind-body”[Title/Abstract] OR “meditative movement”[Title/Abstract]) AND (“Frailty”[Mesh] OR “Frail Elderly”[Mesh] OR frail[Title/Abstract] OR frailty[Title/Abstract] OR pre-frail[Title/Abstract] OR prefrailty[Title/Abstract] OR “pre frail”[Title/Abstract]) AND (“Postural Balance”[Mesh] OR “Accidental Falls”[Mesh] OR balance[Title/Abstract] OR falls[Title/Abstract] OR fall[Title/Abstract] OR “postural control”[Title/Abstract] OR “postural stability”[Title/Abstract] OR “standing balance”[Title/Abstract])	March 2026
Embase	('tai chi'/exp OR 'yoga'/exp OR 'qigong'/exp OR 'tai chi':ti,ab OR yoga:ti,ab OR qigong:ti,ab OR baduanjin:ti,ab OR yijinjing:ti,ab OR 'mind-body':ti,ab OR 'meditative movement':ti,ab) AND ('frailty'/exp OR 'frail elderly'/exp OR frail:ti,ab OR frailty:ti,ab OR 'pre-frail':ti,ab OR prefrailty:ti,ab OR 'pre frail':ti,ab) AND ('postural balance'/exp OR 'accidental fall'/exp OR balance:ti,ab OR fall*:ti,ab OR 'postural control':ti,ab OR 'postural stability':ti,ab OR 'standing balance':ti,ab)	March 2026
Web of Science	TS=(“tai chi” OR “tai ji” OR yoga OR qigong OR baduanjin OR yijinjing OR “mind-body” OR “meditative movement”) AND TS=(frail* OR frailty OR “pre-frail” OR prefrailty OR “pre frail” OR “frail elderly”) AND TS=(balance OR falls OR fall* OR “postural control” OR “postural stability” OR “standing balance”)	March 2026
Cochrane Library	(“Tai Chi” OR yoga OR qigong OR baduanjin OR yijinjing OR “mind-body exercise” OR “meditative movement”) AND (frailty OR frail OR “pre-frail” OR prefrailty) AND (balance OR “postural balance” OR falls OR “postural control”)	March 2026
CNKI	(太极拳 OR 太极 OR 八段锦 OR 易筋经 OR 瑜伽 OR 身心运动 OR 冥想运动) AND (衰弱 OR 衰弱前期 OR 老年衰弱 OR 老年人衰弱) AND (平衡 OR 姿势控制 OR 跌倒 OR 平衡功能)	March 2026
Wanfang Data	(太极拳 OR 八段锦 OR 易筋经 OR 瑜伽 OR 身心运动) AND (衰弱 OR 衰弱前期 OR 老年人) AND (平衡 OR 跌倒 OR 姿势控制)	March 2026
VIP Database	(太极拳 OR 八段锦 OR 易筋经 OR 瑜伽 OR 身心运动) AND (衰弱 OR 衰弱前期) AND (平衡 OR 跌倒 OR 姿势控制)	March 2026

Supplementary Table S1. Search strategies for electronic databases

Outcome	Study design	Risk of bias	Inconsistency	Indirectness	Imprecision	Publication bias	Certainty	Effect estimate (SMD, 95% CI)	Importance
**Overall balance**	RCTs	Serious [a]	Not serious	Not serious	Not serious	Could not be assessed [e]	**Moderate** ⊕⊕⊕○	0.54 (0.17, 0.91)	Critical
**Dynamic balance**	RCTs	Not serious	Serious [b]	Not serious	Serious [c]	Could not be assessed [f]	**Low** ⊕⊕○○	−1.08 (−1.41, −0.76)	Critical
**Static balance**	RCTs	Not serious	Serious [b]	Not serious	Very serious [d]	Could not be assessed [e]	**Very low** ⊕○○○	1.36 (0.43, 2.30)	Critical

Footnotes:

a Most included studies had some concerns regarding blinding of participants and personnel, leading to possible performance bias.

b Substantial unexplained statistical heterogeneity ($I^2 \geq 80\%$) across studies.

c Wide confidence intervals in several subgroup analyses and moderate total sample size.

d Very wide confidence interval and only four studies available, yielding an imprecise estimate.

e Publication bias could not be formally assessed because fewer than 10 studies were available for this outcome; therefore, no downgrade was applied.

f Although 10 studies (12 comparisons) were available, the number was insufficient to reliably assess publication bias using funnel plots or Egger's test; no downgrade was applied.

Supplementary Table S2. GRADE evidence profile
